# Complete genome sequence of *Citrobacter braakii* ASE1 generated by PacBio sequencing

**DOI:** 10.1128/MRA.01000-23

**Published:** 2024-02-14

**Authors:** Thao D. Tran, SangIn Lee, Robert Hnasko, Jeffery A. McGarvey

**Affiliations:** 1 Foodborne Toxin Detection and Prevention Research Unit, Agricultural Research Service, U.S. Department of Agriculture, Albany, California, USA; 2 Produce Safety and Microbiology Research Unit, Agricultural Research Service, U.S. Department of Agriculture, Albany, California, USA; University of Strathclyde, Glasgow, United Kingdom

**Keywords:** *Citrobacter*, biocontrol

## Abstract

We report the complete genome sequence of *Citrobacter braakii* ASE1 generated by the PacBio Sequel II platform. This bacterium was isolated from the soil of a lettuce farm in Salinas, CA, USA, in 2020. The genome consists of a single circular chromosome of 5,021,820 bp with a 52.2% GC content.

## ANNOUNCEMENT

*Citrobacter braakii* is a Gram-negative bacterium in the family *Enterobacteriaceae* (1, 2). It is ubiquitous in the environment and can be found in soil, water, sediment, and the gastrointestinal tracts of animals (1, 3). To elucidate information regarding the suitability of *C. braakii* ASE1 for use as a biocontrol agent, we sequenced its genome.

*C. braakii* ASE1 was isolated from the soil (between rows) of a lettuce farm in Salinas, CA, USA. The soil sample was diluted and blended in phosphate buffer saline without filtration, plated onto Reasoner’s 2A agar plates supplemented with cycloheximide (40 mg/L), and incubated at 25°C for 3 days. A colony was purified by restreaking three times on tryptic soy agar (Oxoid, Hampshire, UK) and cultured in tryptic soy broth at 37°C for 24 h with shaking (200 rpm). The SE1 genome was sequenced using the Pacific Biosciences (PacBio, Menlo Park, CA, USA) Sequel II platform. *C. braakii* ASE1 genomic DNA was extracted by sucrose-Tris with phenol-chloroform cleanup extractions as described previously (4). Five micrograms of genomic DNA was sheared using a G-tube (Covaris, Woburn, MA, USA) at 9,000 rpm (5.4 rcf) in a MiniSpin Plus microcentrifuge (Eppendorf, Enfield, CT, USA) to target 7–12 kb fragments. The SMRTbell genome library was prepared as instructed by the SMRTbell prep kit 3.0 procedure and checklist (PacBio, Menlo Park, CA, USA). The sample was run in one single-molecule real-time (SMRT) cell with 120 pM on-plate concentration, Sequel II binding kit 3.2, diffusion loading type, chemistry 11.0, and HiFi reads.

The ASE1 genome was barcoded using SMRTbell barcoded adapter plate 3.0 (PacBio, Menlo Park, CA, USA), pooled with two other bacterial genomes, and sequenced in one SMRT cell. The PacBio Sequel II produced 100,086,616 raw reads (read *N*50 = 244,153, and subread *N*50 = 7,859). The raw data were demultiplexed using demultiplex barcode analysis (obtained 33,493,448 reads), run through circular consensus sequencing (CCS) application to obtain a Hifireads file, and assembled using Microbial Genome Analysis (SMRT Link v11.1, PacBio, Menlo Park, CA, USA). The CCS analysis produced 1,197,966 HiFi reads (median read quality = Q43, mean read length = 7,553 bp), of which 1,193,293 (99.6%, *N*50 mapped read length = 8,075 bp) were used to assemble one chromosomal contig and circularize without rotating to a particular start gene using SMRT Link v11.1 with Microbial Genome Analysis application. PacBio DNA internal control complex 3.2 was used as an internal sequencing control. Protein-, rRNA-, and tRNA-coding genes were annotated using the NCBI Prokaryotic Genome Annotation Pipeline v6.4 (5). The *C. braakii* ASE1 genome consists of a single circular chromosome of 5,021,820 bp (1,795× coverage; % GC: 52.2%). The genome is predicted to have 4,642 coding sequences, 8 rRNA operons, and 83 tRNAs. The *C. braakii* ASE1 genome has 97% pairwise identity (ref-seq coverage = 96.3%) to *C. braakii* Colony312 (GenBank accession number: CP078598) (LASTZ whole genome alignment, Geneious Prime 2023.0.4). Default parameters were used except where otherwise noted.

## Data Availability

The *C. braakii* ASE1 genome sequence has been deposited in DDBJ/ENA/GenBank under the accession number CP118774, BioProject number PRJNA938151, and BioSample number SAMN33426904. The demultiplexed set of PacBio raw reads for ASE1 is available via SRA under the accession number SRR23675838.
